# Adjuvant treatment of coronary heart disease angina pectoris with Chinese patent medicine: A prospective clinical cohort study

**DOI:** 10.1097/MD.0000000000016884

**Published:** 2019-08-16

**Authors:** Yijia Liu, Zhu Li, Dandan Shen, Yanqi Song, Mengnan Huang, Xiaoxue Xue, Jing Xie, Ziyi Jiao, Shuming Gao, Yilan Xu, Shan Gao, Xianliang Wang, Qiang Xu, Sheng Gao, Chunjie Li, Lin Li, Kaijun Niu, Chunquan Yu

**Affiliations:** a Graduate School; b First Teaching Hospital; c Editorial Department, Tianjin University of Traditional Chinese Medicine; d Second Teaching Hospital, Tianjin University of Traditional Chinese Medicine; e Tianjin Nankai Hospital; f Tianjin Chest Hospital; g Tianjin Medical University, Tianjin Shi, China.

**Keywords:** Chinese patent medicine, coronary heart disease angina pectoris, prospective clinical study

## Abstract

**Background:**

Patients with coronary heart disease (CHD) angina pectoris are in critical condition, which can cause sudden death, myocardial infarction, and other adverse events, and bring serious burden to families and society. Timely treatment should be given to improve the condition. Western medicine treatment of angina pectoris failed to meet the demand of angina symptom control.

**Objective:**

It is hoped that the research method with higher evidential value will be adopted to compare the short-term, medium-term, and long-term effects of Chinese patent medicine combined with conventional western medicine and conventional western medicine alone in the treatment of CHD angina pectoris, so as to tap the clinical efficacy advantages of traditional Chinese medicine (TCM) and provide reliable data support for its clinical application.

**Methods:**

A prospective cohort study was conducted among patients with CHD angina pectoris who were treated with oral Chinese patent medicine and conventional western medicine. The patients were divided into exposed group and nonexposed group according to whether or not the patients with CHD angina pectoris were treated with Chinese patent medicine. The exposed group was treated with TCM combined with conventional western medicine, while the nonexposed group was treated with conventional western medicine alone. Patients need to be hospitalized for 2 weeks as the introduction period and whether to enter the group is determined according to the treatment and medication conditions of the patients. The follow-up time points were 0th, 4th, 12th, 24th, and 48th weeks. The main events and secondary events were used as the evaluation criteria for clinical efficacy of CHD angina pectoris. In the experimental study, we will use strict indicators to detect standard operation procedure for multinomics and bacterial flora detection.

**Conclusion:**

This study will provide evidence for the clinical efficacy advantages of Chinese patent medicine and reliable support for its clinical application through test data.

## 1 Introduction

Coronary heart disease (CHD) is a heart disease caused by myocardial ischemia and hypoxia due to coronary atherosclerosis. Angina pectoris is closely related to coronary artery insufficiency and is common in clinical practice.^[1,2,3]^ Patients with CHD angina pectoris are in critical condition, which can cause sudden death, myocardial infarction, and other adverse events, and bring serious burden to families and society. Timely treatment should be given to improve the condition.^[2,3,4,5]^ Clinical attention has long been paid to the treatment of CHD angina pectoris. Western medicine for angina pectoris mainly uses β-receptor blocker, calcium channel blocker, angiotensin converting enzyme inhibitor, angiotensin II receptor antagonist, etc.^[6]^ It has good clinical effect, but the demand for angina symptom control is still unsatisfied.^[7]^

As a unique medical resource in China, traditional Chinese medicine (TCM) plays an important role in disease treatment and health services. Chinese patent medicine is made of Chinese herbal medicines as raw materials and processed into various dosage forms of Chinese traditional medicine products, including pills, powders, granules, pastes, capsules, Chinese traditional medicine injections, aerosols, etc. Its advantages are ready availability, adaptability to urgent need, convenient storage, easy carrying, and saving decocting process, etc, and eliminating peculiar smell and adverse stimulation when taking TCM decoction.^[8,9,10]^ According to statistics, the number of Chinese patent medicines in the 2012 edition of the basic medicine catalog has increased to 203, accounting for 39%, of which 23 are Chinese patent medicines for the treatment of cardiovascular diseases.^[11]^ Eight kinds of commonly used cardiovascular Chinese patent medicines and 28 kinds of antiangina Chinese patent medicines are included in New Pharmacology.^[12]^ In recent years, many studies have shown that Chinese patent medicine has achieved certain curative effect in the dialectical treatment of angina pectoris, which has the advantages of good effect and few adverse reactions.^[13,14,15,16,17]^ However, Chinese patent medicine is difficult to match western medicine with large-scale clinical trials in treatment, health education, and publicity. Therefore, a research method with higher evidence value-clinical cohort study is needed to conduct evidence-based research on it.

The results of cohort study belong to class II evidence-based medicine, and its research evidence is second only to randomized controlled trials (RCTs). However, cohort study has its advantages such as long time, large sample, and so on. Cohort study design can follow the characteristics of individualized diagnosis and treatment of TCM and dynamic changes of treatment schemes, and take Chinese patent medicine as a factor to comprehensively evaluate the curative effect of Chinese patent medicine on CHD angina pectoris.^[18]^ In this study, prospective cohort study was used to study the clinical efficacy of Chinese patent medicine in the adjuvant treatment of CHD angina pectoris, so as to enrich the evidence of efficacy evaluation of Chinese medicine. Establish innovative methods and techniques for the evaluation of clinical efficacy of TCM, and then promote its deeper research and application.

## 2 Method

**Figure 1:**
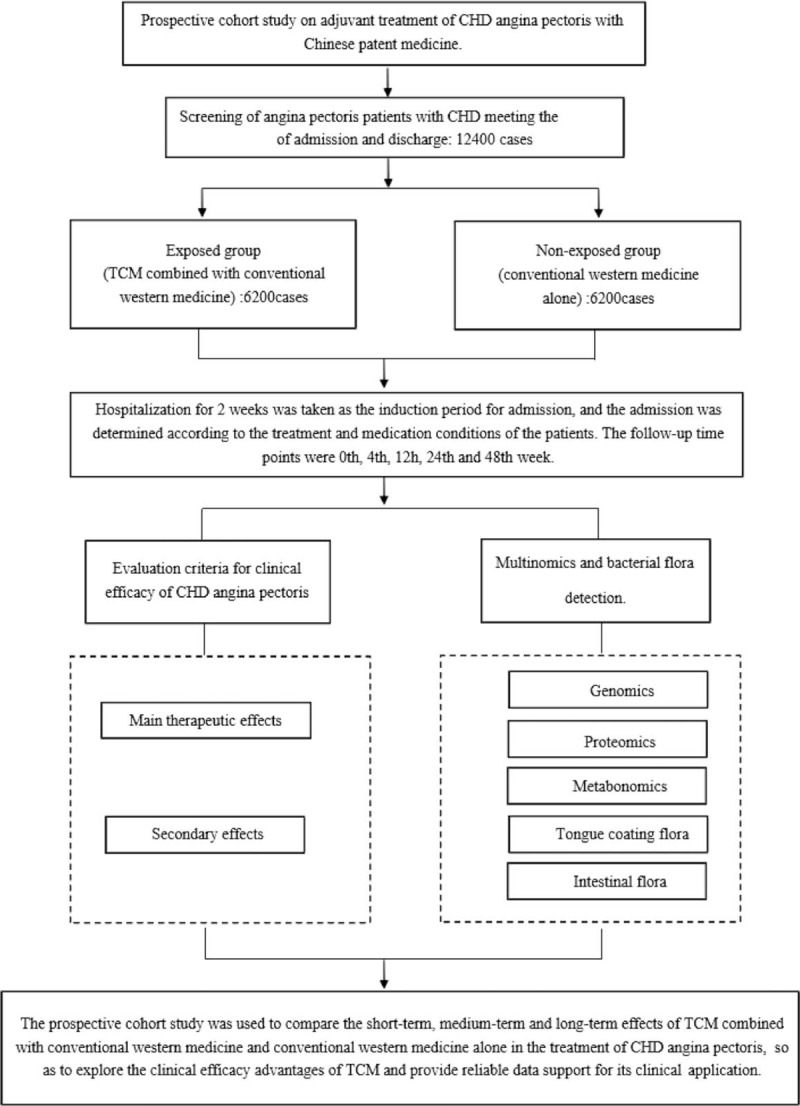
Prospective cohort study of Chinese medicine in the treatment of coronary heart disease (CHD) angina pectoris. TCM = traditional Chinese medicine.

A prospective cohort study was conducted among patients with CHD angina pectoris who were treated with oral Chinese patent medicine and conventional western medicine. The patients were divided into exposed group and nonexposed group according to whether or not the patients with CHD angina pectoris were treated with Chinese patent medicine. The exposed group was treated with TCM combined with conventional western medicine, while the nonexposed group was treated with conventional western medicine alone. Patients need to be hospitalized for 2 weeks as the introduction period and whether to enter the group is determined according to the treatment and medication conditions of the patients. The follow-up time points were 0th, 4th, 12th, 24th, and 48th weeks. The main events, secondary events and adverse events were used as the evaluation criteria for clinical efficacy of CHD angina pectoris. In the experimental study, we will use strict indicators to detect standard operation procedure for multigroup and bacteria detection. It is hoped that the research method with higher evidential value will be adopted to compare the short-term, medium-term, and long-term effects of Chinese patent medicine combined with conventional western medicine and conventional western medicine alone in the treatment of CHD angina pectoris, so as to tap the clinical efficacy advantages of TCM and provide reliable data support for its clinical application. The technical route is shown in Figure 1.

### 2.1 Study site

The cooperative network of CHD research medical units formed by the research team includes 5 hospitals, including the First Affiliated Hospital of Tianjin University of TCM, the Second Affiliated Hospital of Tianjin University of TCM, Tianjin Chest Hospital, Tianjin Nankai Hospital, and Tianjin Medical University General Hospital.

### 2.2 Qualified research subject

#### 2.2.1 Diagnostic criteria of western medicine

Diagnostic criteria for CHD angina pectoris: According to the guidelines for the diagnosis of CHD angina pectoris formulated by the American Heart Association and the Heart Association (ACC/AHA) in the Guidelines for the Treatment of Chronic Stable Angina Pectoris (2007) and the Guidelines for the Management of Patients with Unstable Angina Pectoris and Non-ST-Elevation Myocardial Infarction (2011), and referring to the Guidelines for the Diagnosis and Treatment of Chronic Stable Angina Pectoris in China in 2007, and the Guidelines for the Diagnosis and Treatment of Unstable Angina Pectoris and Non-ST-Elevation Myocardial Infarction in China in 2007, diagnostic criteria are formulated by Chinese Society of Cardiology, Chinese Journal of Cardiology. And the Chinese Medicine Research Association of China and Western Medicine combined with the Cardiovascular Disease Prevention and Rehabilitation Committee established the Standard for the Consensus of Integrative Medicine for Integrative Medicine for the Treatment of Coronary Heart Disease (2019).

Diagnosis can be made by meeting any one or more of the following:

1. The definite history of old myocardial infarction.
2. Coronary angiography or computed tomography angiography examination showed that the diameter of at least 1 main branch of coronary artery was ≥50%.
3. Patients with ST-segment depression or T-wave inversion after coronary revascularization (percutaneous transluminal coronary intervention [PCI] or coronary artery bypass grafting [CABG]).

The diagnosis of unstable angina pectoris of CHD refers to the Guidelines for Diagnosis and Treatment of Unstable Angina Pectoris and Non-ST-Segment Elevation Myocardial Infarction issued by Chinese Society of Cardiology and Chinese Journal of Cardiology in 2007, including the following subtypes:

1. Resting angina pectoris: Angina pectoris attack at rest, and the duration is usually more than 20 minutes.
2. Initial angina pectoris: New angina pectoris occurred within 1 month, which may be characterized by spontaneous and exertional episodes, with pain ranking above grade III.
3. Aggravated exertional angina pectoris: Previous history of angina pectoris, aggravation of angina pectoris in the past month, frequent attacks, prolonged time or reduced pain closure (angina pectoris grade at least increased by grade I, or at least reached grade III).
4. Variant angina pectoris: Usually spontaneous. It is characterized by transient ST segment elevation, most of which spontaneously relieves and does not develop into myocardial infarction, but a few of which can develop into myocardial infarction. Atherosclerotic plaque leads to local endothelial dysfunction and coronary artery spasm, which are the causes of the disease. Nitroglycerin and calcium antagonist can relieve it.

#### 2.2.2 Criteria for differentiation of symptoms and signs in TCM

The TCM syndrome differentiation reference standard refers to the 2002 edition of the Guiding Principles for Clinical Research of TCM (New Drugs), the internal medicine of TCM (the 7th edition of teaching materials), and the revision of the consensus of experts in TCM diagnosis and treatment of chest pain after PCI in 2014.

#### 2.2.3 Inclusion criteria

The inclusion criteria were as follows:

1. Voluntary participation, understanding, and signing of informed consent form.
2. The age ranged from 35 to 75 years old and gender is not limited.
3. For patients with angina pectoris of CHD in the inpatient department or outpatient department of cardiology, the diagnosis of angina pectoris conforms to the relevant guidelines issued by ACC/AHA, Chinese Society of Cardiology and Chinese Journal of Cardiology.
4. For patients who meet the standard of drug therapy, the exposed group is treated with Chinese patent medicine combined with conventional western medicine, while the nonexposed group is treated with conventional western medicine alone.

#### 2.2.4 Exclusion criteria

The exclusion criteria were as follows:

1. Patients who are combined with other cardiac diseases, neurosis, climacteric syndrome, hyperthyroidism, cervical spondylosis of spinal cord or vertebral artery type, gastroesophageal reflux disease or hiatal hernia, and other diseases that may cause chest pain.
2. Angina pectoris patients with serious and uncontrollable symptoms, suffering from acute myocardial infarction, heart failure, myocarditis, cardiomyopathy, severe heart valve diseases, liver failure or renal failure, malignant tumor, severe metabolic diseases, and other major diseases.
3. Women who are pregnant or lactating or women are in childbearing age with fertility requirements.
4. Patients with a mental disorder or cognitive dysfunction.
5. Patients who have participated in other trials within the past 3 months.
6. Patients with a history of allergy or with a known allergy to the study drug.
7. Patients who are expected to poor compliance and unpossible regular visits.
8. Patients with no current address or incomplete current address without contact telephone number.
9. Patients who are unable to participate in the study, as judged by the investigator.

### 2.3 Sample size calculation

**Figure 2:**
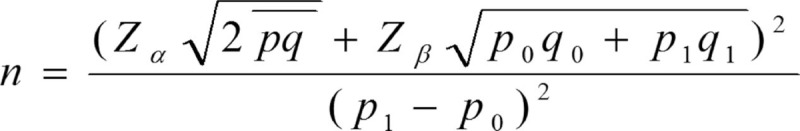
The sample size calculation formula.

The sample size calculation formula is shown in Figure 2. The expected incidence rates of exposed group and nonexposed group were obtained through literature review,^[18,19]^ *P*_1_ = 0.072, *P*_0_ = 0.059, *q* = 1 − *p*, *Z*_*α*_ and *Z*_*β*_ were the areas under the standard normal distribution, and the table was found to obtain *Z*_*α*_ = 2.58, *Z*_*β*_ = 1.64. Substituting the above values into the formula can be obtained, and the sample size of each group is about 5677 cases. Control the rate of loss of follow-up within 10%, mainly to ensure a certain professional representation. There were about 6200 cases in each group, and the total sample size of this study was about 12,400.

### 2.4 Therapeutic drug

#### 2.4.1 Oral Chinese patent medicine commonly used in CHD angina pectoris

Shexiang Baoxin Pill, Tongxinluo Capsule, Tongmai Yangxin Pill, Xuefu Zhuyu Capsule, and Qishen Yiqi Dropping Pill.

#### 2.4.2 Conventional western medicine for CHD angina pectoris

Antiplatelet drugs, statins, beta blockers, calcium channel blockers, angiotensin converting enzyme inhibitor, angiotensin II receptor antagonists, and so on.

### 2.5 Adverse reactions

In this study, the adverse reactions of drugs (Chinese patent medicine and conventional western medicine) studied are detailed in the drug instructions.

### 2.6 Regulations on combined use of drugs

Patients with CHD angina pectoris can be treated with the original scheme before admission during the study period, and the admission period is 2 weeks, so that the patient's condition is stable and meets the admission criteria.

During the study period, for patients with hypertension, diabetes, hyperlipidemia, and other diseases, the antihypertensive, hypoglycemic, and lipid-lowering treatment schemes before admission can be maintained. The name, dosage, and usage of the drugs are recorded in detail in the study medical records. It is forbidden to adjust the dosage and usage of β-receptor blockers, calcium ion antagonists, and other drugs that affect the curative effect of CHD angina pectoris.

### 2.7 Follow-up time point

Patients need to be hospitalized for 2 weeks as the introduction period and whether to enter the group is determined according to the treatment and medication conditions of the patients. The follow-up time points were 0th, 4th, 12th, 24th, and 48th weeks. The main curative effect, secondary curative effect, and concomitant medication changes of patients were observed.

### 2.8 Information collection

#### 2.8.1 Demographic data

Including the patient's gender, age, nationality, occupation, height, weight, and body mass index.

#### 2.8.2 TCM syndrome types of CHD angina pectoris

The TCM syndrome type is derived from the diagnosis of the existing cases. If there is no syndrome diagnosis, the 2 trained physicians with more than 5 years of practicing qualifications will judge each other. If there is any dispute, they will be submitted to the superior doctor for final review.

#### 2.8.3 Western medicine typing of CHD angina pectoris

Western medicine classification of CHD angina is derived from the diagnosis of existing cases. If there is no classification diagnosis, 2 of the trained physicians with more than 5 years of practicing qualifications judge each other. If there is any dispute, they will be submitted to the superior doctor for final review.

#### 2.8.4 Evaluation criteria for clinical efficacy of CHD angina pectoris

1. Main events: The incidence of cardiovascular events includes acute myocardial infarction, PCI or CABG.
2. Secondary events
  (a) All causes of death.
  (b) Readmission due to cardiovascular events.
  (c) Seattle Angina Questionnaire (SAQ): The degree of pain in angina pectoris, the frequency and duration of acute paroxysmal angina pectoris, etc.
  (d) Routine admission examination: Carotid artery color Doppler ultrasound, cardiac color Doppler ultrasound, electrocardiogram, transcranial color Doppler ultrasound, chest radiograph, and other imaging examinations; Clinical biochemical tests such as troponin, brain natriuretic peptide, emergency 7 items, myocardial enzyme, D-dimer, coagulation four items, hematuria and stool routine, etc.
3. Adverse events:
  (a) Produce toxic reactions (such as severe gastrointestinal reactions, gingival bleeding, etc).
  (b) Anaphylactic shock.
  (c) Liver and kidney function damage caused by TCM.

#### 2.8.5 Histology and flora detection

Samples with the same number of cases in exposed group and nonexposed group were respectively selected for testing, in strict accordance with genomics, proteomics, metabolomics, intestinal flora, and tongue coating flora detection methods for analysis. The following points should be paid attention to in the process of index detection:

1. Select patients with good compliance to ensure timely medication and keep a light diet during medication to avoid interference from alcohol and carbonated beverages.
2. Determination of clinical biochemical indexes fasting for 12 hours, and within 24 hours should not be intense sports. Blood should be collected in the decubitus position, and rest in the decubitus position for 1.5 to 2 hours before blood collection. Morning urine and middle urine should be taken. Feces avoid the pollution of urine and toilet walls, collect fresh feces from the middle and rear part of the excretion, and dig up the feces from the inner side of the middle part as samples.
3. Sample collection is limited to 1 month and the collected samples are transported in a centralized way. Biologic samples should be stored in a refrigerator at −80°C. Theoretically, all tested samples should be analyzed within 6 months.

#### 2.8.6 Information collection at different time points

1. 0th week: Collect demographic data, TCM syndromes of CHD angina pectoris, western medicine classification of CHD angina pectoris, SAQ in main curative effect and secondary curative effect, routine admission examination, genomics, proteomics, metabolomics, intestinal flora, and tongue coating flora.
2. 4th week: SAQ, routine admission examination, adverse events, genomics, proteomics, metabolomics, intestinal flora, and tongue coating flora were tested for primary events and secondary events.
3. 12th week: SAQ, routine admission examination, adverse events, genomics, proteomics, metabolomics, intestinal flora, and tongue coating flora were tested in the primary events and secondary events.
4. 24th week: Detect the main curative effect, death in secondary curative effect, readmission due to cardiovascular events and adverse events.
5. 48th week: Check the main curative effect, death in secondary curative effect, readmission due to cardiovascular events and adverse events (Table 1).

**Table 1:**
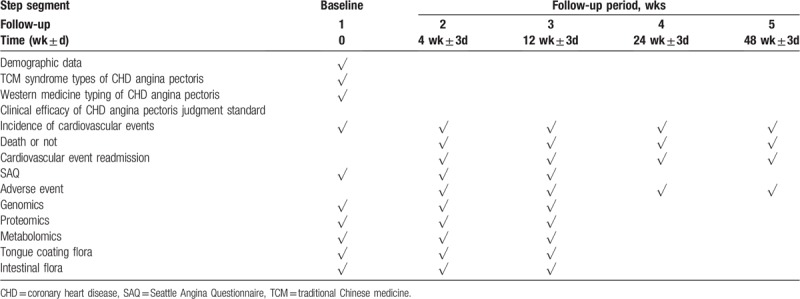
Patient-related information collection process.

### 2.9 Information collection methods

In 0th week, relevant patient information was collected by consulting medical records, medical record systems, or interviews. In the 4th, 12th, 24th, and 48th weeks, interviews, telephone calls, WeChat (which can establish WeChat groups for patients and be managed in a unified way), or outpatient follow-up system are conducted to record the occurrence time and data of follow-up indicators and establish a statistical database.

### 2.10 Data management

#### 2.10.1 Original data management and archiving

Cardboard case report form (CRF) is adopted, and all CRFs will come into effect after being reviewed and signed by researchers. All CRFs and CRF modification traces are retained. Each test report of the subject was pasted at the designated position.

#### 2.10.2 Data entry and storage

The data are entered into the computer by 2 persons and 2 persons using EpiData 3.1 database, and the data are entered into the computer by 2 trained professionals, respectively. The database is established by EpiData 3.1 for data entry. According to the research content, a data entry guide will be prepared and data entry personnel will be specially trained before data entry. Data entry personnel enter data according to the entry guide.

#### 2.10.3 Writing of data verification and answers to questions

The entered data will be checked one by one according to the Data Verification Plan (SAP). SAP needs the signature and approval of the data administrator and statistician.

Verification methods include systematic verification and manual medical verification. Systematic verification mainly uses SAS 9.3 software to write verification procedures and generate data clarification form (DCF). All data query contents found through manual medical verification and systematic verification will be input into the project data query database, and the data management unit will make a formal DCF. DCF shall be sent to the clinical research supervisor after being checked and found to be correct. After re-examination by the inspectors, they will be handed over to researchers in various research centers for examination and answer. Only after receiving the DCF with the signature and date of the researcher can it be confirmed that the DCF has been answered and the database can be modified. The original DCF will be stored in the special filing cabinet for the project.

**Figure 3:**
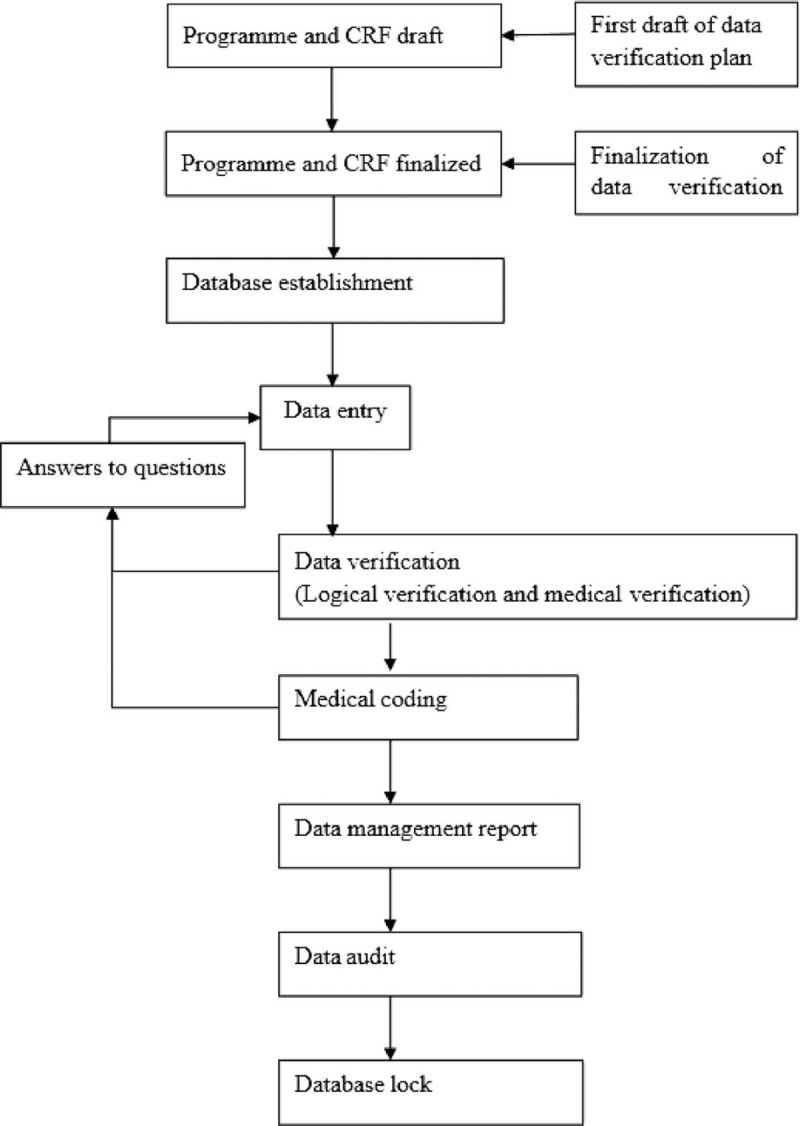
Data management general process. CRF = case report form.

Any changes to the database will be recorded, recording when, by whom, and for what reason (Fig. 3).

### 2.11 Statistical analysis

#### 2.11.1 Statistical analysis of data

Before the statistical analysis of clinical test data, the statistical analysis personnel shall formulate a statistical analysis plan, specify detailed statistical analysis steps, and carefully check, clean up, and confirm the clinical test data. The analysis content includes case elimination and shedding analysis, checking the number of completed cases, elimination and shedding in each center, descriptive statistical analysis, listing the frequency distribution of each variable, comparison of baseline data of clinical characteristics before treatment, analysis of concomitant medication, analysis of curative effect, safety evaluation and analysis of missed visits, etc. All statistical analysis processes are programmed and archived for future reference.

#### 2.11.2 Main statistical analysis methods

Descriptive analysis and inferential analysis of clinical features before and after treatment were as follows:

1. Metrologic data: Comparative analysis before and after treatment between groups or within groups. First, normal test is carried out on the distribution of variables.
  (a) When obeying normal distribution, statistical description is expressed by mean number, standard deviation, number of cases, minimum value and maximum value, etc. Statistical method is *t* test or variance analysis.
  (b) Nonnormal distribution. Statistical description is expressed by median, upper quartile (Q1), lower quartile (Q3), 95% confidence interval, etc. Statistical method is analyzed by rank sum test (Wilcoxon method).
  (c) If the influence of the center or other factors is considered, covariance analysis is used.
2. Counting data: Descriptive statistical analysis is made by frequency table, percentage, or composition ratio. Comparative analysis before and after intra-group or intra-group treatment was performed by Chi-squared test and Fisher exact probability method. At the same time, relative risk was calculated to illustrate the correlation strength between exposure and outcome.
3. Grade data: Rank sum test (Mann–Whitney method) was used for comparison between groups.
4. Analysis of influencing factors of curative effect: Regression analysis is adopted, including age, sex, treatment, and other factors that may affect the judgment of curative effect.
5. The incidence rate and median time of cardiovascular events were analyzed by Kaplan–Meier method, and Cox regression analysis was used to process truncated data of cardiovascular events caused by missed visits.
6. Stratified analysis: Compare the stratified curative effects by drug usage and drug treatment duration. Baseline comparison test level *α* = 0.05, and efficacy comparison test level *α* = 0.05 between groups. General statistical tests adopt bilateral tests, and those with *P* <.05 are considered to have statistical significance.

#### 2.11.3 Safety analysis

The safety analysis of each group of cases is mainly based on descriptive statistics, including the incidence rate of adverse events and abnormal changes in the specific description of adverse events and their relationship with drug administration.

#### 2.11.4 Ethical principles

The subjects were voluntary subjects from 5 centers, all of whom independently signed informed consent forms and put them into CRF inserts. Ethical review prior to the start of clinical research, research on this topic must be reviewed by the ethics committee of each center's research unit. The subject has passed the ethical review by the ethics Committee of Tianjin University of TCM. The approval document number for the ethics review is TJUTCM-EC20190007. It was registered with the Chinese Clinical Trial Registry on July 14, 2019 with registration number ChiCTR-1900024532. Registered at the Clinical Trials.gov on July 16, 2019, registration number is NCT04022031.

## 3 Discussion

The results of cohort study belong to Class II evidence-based medicine and have advantages such as long-term, large sample, and so on. In the prospective cohort study, we can directly obtain the 1st-hand data on exposure and outcome, so the bias of the data is small. On the basis of the National Basic Research Program of China (973 projects), this study has carried out a rigorous and meticulous design of this plan and has the following advantages:

1. Fully collect important clinical-related results and the main indicators, secondary indicators and adverse event reports included in the test.
2. Strict concomitant medication regulations: As patients with CHD are often complicated with hypertension, hyperlipidemia, and other diseases, or undergo PCI surgery, they need to take antihypertensive, antiplatelet agglutination, and other drugs for a long period of time. This study has strict regulations on concomitant medication, reflecting the rigor of the design.
3. Accurate methodology design to reduce the risk of bias: Before statistical analysis of clinical test data, statistical analysts shall formulate statistical analysis plans, specify detailed statistical analysis steps, and carefully check, clean up and confirm clinical test data.
4. Strict quality control in clinical research: Due to different clinical units, to prevent the differences in the process of collecting samples, the research group dispatched uniformly trained supervision and inspection personnel to carry out off-site and on-site supervision and inspection work on clinical units to ensure the strictness of the patients enrolled. The filling of clinical scales is also carried out after training, and professional personnel supervise and verify the data on site. The later research group uniformly conducts 2 rounds of verification on the data to ensure the accuracy of the original data.
5. Unified sample collection and testing: Collected blood and urine samples are collected, transported, processed, and stored in a unified way. Biochemical indexes, liquid chips, and expression spectrum chips are all detected by professional companies to ensure the unity of the detection process.

Due to the complexity of clinical research, although the research scheme has strict regulations on possible problems, it may still affect some research results. The clinical research plan of this kind of research will be further improved in the later period according to the actual research situation. This plan will comprehensively explore the clinical efficacy advantages of Chinese patent medicine in adjuvant treatment of CHD angina pectoris, and provide reliable data support for its clinical application.

## Author contributions

**Conceptualization:** Chunquan Yu, Lin Li, Kaijun Niu.

**Investigation:** Yanqi Song, Mengnan Huang, Xiaoxue Xue, Jing Xie, Ziyi Jiao.

**Methodology:** Chunquan Yu, Lin Li, Kaijun Niu.

**Supervision:** Chunquan Yu, Shuming Gao, Yilan Xu, Shan Gao, Xianliang Wang, Qiang Xu, Sheng Gao, Chunjie Li, Lin Li, Kaijun Niu.

**Validation:** Chunquan Yu, Shuming Gao, Yilan Xu, Shan Gao, Xianliang Wang, Qiang Xu, Sheng Gao, Chunjie Li, Lin Li, Kaijun Niu.

**Writing – original draft:** Yijia Liu, Zhu Li, Dandan Shen.

**Writing – review & editing:** Yijia Liu, Zhu Li, Dandan Shen.
